# Objective assessment of graft clarity and recurrence after penetrating keratoplasty for granular, lattice and macular corneal dystrophy using scheimpflug densitometry

**DOI:** 10.1007/s00417-025-07050-x

**Published:** 2025-12-15

**Authors:** Tim Berger, Berthold Seitz, Elias Flockerzi, Albéric Sneyers, Shady Suffo, Loay Daas

**Affiliations:** Department of Ophthalmology, Saarland University Medical Center, Kirrberger Str, Homburg/Saar, D-66424 Germany

**Keywords:** Penetrating keratoplasty, Corneal dystrophy, Corneal densitometry, Corneal surgery, Corneal opacity, IC3D

## Abstract

**Purpose:**

To evaluate long-term outcomes and recurrence patterns following penetrating keratoplasty (PKP) in granular (GCD), lattice (LCD), and macular corneal dystrophy (MCD), using Scheimpflug-based corneal densitometry (grayscale units–GSU) as an objective tool to assess graft clarity.

**Methods:**

In this retrospective single-center study, 99 eyes of 59 patients with GCD (*n* = 38), LCD (*n* = 30), or MCD (*n* = 31) were analyzed. A total of 112 PKPs, including 74 excimer laser-assisted PKPs, were evaluated. Clinical examinations included visual acuity, slit-lamp evaluation, and corneal tomography using the Pentacam HR. Clinical recurrence was defined as the appearance of dystrophy-specific changes within the graft. Corneal densitometry was assessed in the anterior, central, and posterior layers and the total corneal thickness at 0–2 mm and 2–6 mm zones. Follow-up ranged from 6 weeks to more than 5 years (5y+) postoperatively.

**Results:**

A significant postoperative improvement in BCVA was observed in eyes with GCD and MCD, with sustained visual gains up to 4y and 5y postoperatively, respectively. GCD demonstrated the earliest and highest clinical recurrence rate, with all grafts affected by 5y. LCD showed delayed recurrence from 4y onward, while MCD did not show any recurrence within 5y. Corneal densitometry revealed a progressive increase in GSU in GCD and LCD, particularly in the anterior (GCD:5y / LCD:5y+) and central layers (GCD:4y / LCD:5y+). MCD showed stable GSU values throughout follow-up. Linear regression analysis showed the strongest GSU increase in LCD (slope = 1.65, R²=0.47) and GCD (slope = 0.94, R²=0.14), particularly in the anterior 0–2 mm zone. MCD showed minimal change across all layers and diameters.

**Conclusion:**

Scheimpflug-based corneal densitometry enables objective, layer-specific monitoring of graft clarity and recurrence after PKP. Recurrence rates differ significantly among dystrophy subtypes, highlighting the clinical utility of densitometry in tailoring follow-up strategies, particularly in GCD and LCD with high risk of recurrence.

**Supplementary Information:**

The online version contains supplementary material available at 10.1007/s00417-025-07050-x.

## Key Messages

**What was known:**

- *Penetrating keratoplasty (PKP) is a well-established treatment for various stromal corneal dystrophies, including granular (GCD), lattice (LCD), and macular corneal dystrophy (MCD), with differing recurrence and graft survival rates.*

**What this paper adds:**

- *Scheimpflug-based corneal densitometry allows for objective, layer-specific quantification of graft clarity and disease recurrence following PKP in epithelial-stromal TGFBI corneal dystrophies and stromal corneal dystrophies.*
- *Corneal densitometry effectively differentiates recurrence characteristics among dystrophy subtypes, with progressive increases in densitometry values observed in GCD and LCD, particularly in the anterior and central stromal layers.*
- *Densitometry changes precede or coincide with clinically observed recurrence, underscoring its potential as a sensitive biomarker for subclinical graft changes and a valuable adjunct in postoperative surveillance.*

**Supplementary Information:**

The online version contains supplementary material available at 10.1007/s00417-025-07050-x.

## Introduction

Corneal dystrophies historically represent a heterogeneous group of hereditary, bilateral, and non-inflammatory disorders affecting various layers of the cornea. These conditions typically progress slowly and are not associated with systemic diseases. The International Committee for the Classification of Corneal Dystrophies (IC3D) categorizes them into epithelial and subepithelial corneal dystrophies, epithelial-stromal *TGFBI* corneal dystrophies, stromal corneal dystrophies, and endothelial corneal dystrophies [1, 2].

Epithelial-stromal *TGFBI* corneal dystrophies are characterized by autosomal dominant mutations in the *TGFBI* (*transforming growth factor beta-induced*) gene, leading to the development of several clinically distinct corneal dystrophies, including lattice corneal dystrophy (LCD) or granular corneal dystrophy (GCD), which is further subdivided into type 1 (GCD1) and type 2 (GCD2). *TGFBI* is located on chromosome 5q31 and encodes transforming growth factor beta-induced protein (TGFBIp), which is an extracellular matrix protein and is secreted by corneal epithelium and keratocytes [3, 4]. In contrast, stromal corneal dystrophies, such as macular corneal dystrophy (MCD, 16q22, *carbohydrate sulfotransferase 6*), primarily affect the stromal layers of the cornea, where pathological deposits and structural alterations are predominantly localized while the epithelium is spared [5–7].

In advanced stages, GCD, LCD, and MCD might demonstrate deep or diffuse stromal involvement, necessitating surgical interventions such as deep anterior lamellar keratoplasty (DALK) or penetrating keratoplasty (PKP). The recurrence of deposits in corneal grafts varies significantly depending on the specific corneal dystrophy. Generally, epithelial-stromal *TGFBI* corneal dystrophies tend to recur earlier, whereas stromal corneal dystrophies typically demonstrate very late recurrences, if at all [2, 8].

Currently, there is no generally accepted definition of corneal dystrophy recurrence following corneal transplantation. Defining recurrence after keratoplasty remains challenging, as early-stage manifestations may be clinically subtle, subject to individual interpretation, and influenced by diagnostic limitations. Moreover, the variability of recurrence characteristics among different corneal dystrophies further complicates the assessment.

An objective evaluation of surgical outcomes can be achieved through corneal densitometry, which quantitatively assesses corneal transparency. Scheimpflug corneal tomography with densitometry enables precise optical analysis by measuring the degree of light transmission and backscatter within the cornea. A decrease in transparency results in increased light backscatter. By quantifying backscattered light, densitometry provides an objective measure of corneal clarity, enhancing the accuracy of recurrence detection [9].

Despite its potential, corneal densitometry has not yet been widely used to systematically assess recurrence patterns after PKP in different types of corneal dystrophies. Given the varying recurrence profiles of GCD, LCD, and MCD, a structured, quantitative follow-up tool is of particular clinical interest. The purpose of this study was to evaluate the long-term morphologic and functional outcomes following PKP, with a specific focus on recurrence detection using Scheimpflug-based corneal densitometry.

## Patients and methods

### Study group

This single-center retrospective study included a total of 99 eyes from 59 patients diagnosed with GCD (*n* = 38), LCD (*n* = 30), and MCD (*n* = 31). A total of 112 PKPs were analyzed comprising 46 for GCD, 34 for LCD, and 32 for MCD.

Of these, 74 eyes underwent primary or repeat excimer laser-assisted PKP (Excimer-PKP) at the Department of Ophthalmology, Saarland University Medical Center, Homburg/Saar, Germany, between 2011 and 2024. The overall number of PKPs also included 38 externally performed procedures, provided that a minimum graft survival of at least five years (5y+) was documented. For these externally performed PKPs, the underlying corneal dystrophy was confirmed using available clinical records (including examination of the fellow eye), slit-lamp photography, and, when applicable, genetic testing, ensuring accurate diagnostic verification prior to inclusion. These externally preoperated eyes were included exclusively for the analysis of the long-term postoperative course, with a particular emphasis on recurrence patterns following PKP. The follow-up period ended in the event of repeat corneal surgery or if the patient no longer appeared for follow-up examinations. Exclusion criteria were corneal endothelial decompensation and systemic or ocular diseases that impair vision.

The study was approved by the local ethics committee of Saarland (*No. 53/24*) and adhered to the ethical principles outlined in the Declaration of Helsinki for medical research involving human subjects.

### Surgery

Preoperative assessment of corneal deposits using anterior segment optical coherence tomography (AS-OCT, CASIA2, Tomey Corp., Nagoya, Japan) guided the indication for PKP. When deposits were predominantly confined to the subepithelial or anterior stromal layers, phototherapeutic keratectomy (PTK) was preferred. This decision was based solely on the depth and distribution of the corneal opacities, as PTK is generally indicated when no more than 10–20% of the anterior stroma is affected [10]. However, in the cases analyzed in this study, the corneal dystrophies were generally more advanced, with deposits extending into the deeper stromal layers, making PTK unsuitable and necessitating PKP.

Surgery was performed under general anesthesia. Nonmechanical trephination was performed using the 193 nm excimer laser (Schwind Amaris 1050 RS, Schwind eye-tech-solutions, Kleinostheim, Germany) along metal masks with eight orientation notches [11]. For donor trephination from the epithelial side using the 193 nm excimer laser, a circular, round metal aperture mask was positioned on a corneoscleral button (16-mm diameter) fixed in an artificial anterior chamber under microscopic control. The pressure within the artificial anterior chamber was adjusted to 20 mmHg by attaching it to an infusion system. After perforation, the remaining stromal lamellae and Descemet’s membrane were cut with a curved corneal microscissors. For excimer laser-assisted trephination of the recipient cornea, a corresponding circular, round metal mask was used. To complete trephination, the remaining deep stromal lamellae and Descemet’s membrane were cut with a curved corneal microscissors. In all patients, a peripheral iridotomy was performed at the 12-o’clock position. After temporary fixation of the donor button in the recipient bed with 8 interrupted sutures, a permanent wound closure was achieved by a 16-bite double-running cross-stitch suture (10 − 0 nylon) according to Hoffmann. The first running suture was removed after 12 months, while the second suture was removed after 18 months.

### Follow-up and measurement technique

Follow-up examinations after PKP were scheduled after 6 weeks (6w), 1 year (1y), 3 months after removal of the first suture (1st suture out), 3 months after removal of the second suture (2nd suture out) and then annually up to more than 5 years (5y+).

For the additional assessment of dystrophy recurrences, eyes in which PKP had been performed at least 5y previously at an external clinic were also included. All patients were clinically examined and underwent comprehensive slit-lamp biomicroscopy including funduscopy. The minimum follow-up period after Excimer-PKP was 6w. Tomographic measurements were conducted at each follow-up examination using the Pentacam HR (Oculus Optikgeräte GmbH, Wetzlar, Germany). The Pentacam HR uses a blue light-emitting diode light source (wavelength 475 nm, scanning depth 6 mm) and a Scheimpflug camera which rotates 360 degrees (100 slit images in 2 s) for generating cross-sectional images. The calibration was carried out in accordance with the manufacturer’s instructions. Only measurements with an acceptable quality score were included in the analysis. Due to the retrospective nature of the study, each measurement was performed once. The following parameters (in diopters, D) of the anterior corneal surface were evaluated: keratometry of the flat (K1) and steep (K2) meridian, mean keratometry (Kmean), maximum keratometry (Kmax), astigmatism. Evaluation of corneal pachymetry (in µm) included the central corneal thickness (CCT) and the thinnest corneal thickness (TCT). Corneal densitometry was assessed in grayscale units (GSU), which range from 0 (no opacity) to 100 (completely opaque). Corneal densitometry was only assessed in the 0–2 mm zone and the 2–6 mm zone as the graft-host junction might influence the measurements at higher diameters. The evaluation was carried out separately for the anterior corneal layer (anterior 120 μm), the center layer (between the anterior and posterior area), the posterior corneal layer (posterior 60 μm) and the total corneal thickness.

### Statistical analysis

Microsoft Excel (Microsoft Corp., Redmond, WA, USA) was used for data collection and GraphPad Prism 9.0 (GraphPad Software Inc., Boston, MA, USA) for statistical evaluation. Data were expressed as mean ± standard deviation (SD) and range. Decimal acuity was converted to equivalent logMAR (logarithm of the minimum angle of resolution) visual acuity.

Since this study includes data from both eyes of an individual, a mixed model analysis was performed to adjust for the potential correlation between the two eyes. Furthermore, this statistical method was chosen to account for the absence of follow-up measurements at certain time points [12, 13]. Post hoc comparisons were performed using Tukey’s test. P-values ≤ 0.05 were considered statistically significant. Statistical analyses were limited to comparisons of measurements within the same group. Linear regression analyses were performed to assess trends over time.

## Results

This study analyzed 99 eyes from 59 patients (29 males, 30 females) diagnosed with GCD (*n* = 38), LCD (*n* = 30) and MCD (*n* = 31). The mean age at time of primary PKP was 53.3 ± 12.2 years for GCD (range: 31–78 years), 54.5 ± 12.3 years for LCD (range: 28–75 years) and 33.7 ± 9.8 years for MCD (range: 19–59 years).

A total of 112 PKPs were evaluated, comprising 46 grafts of GCD, 34 of LCD, and 32 of MCD. Of these, 38 PKPs (GCD: *n* = 19/LCD: *n* = 13/MCD: *n* = 6) had been performed at external institutions and exhibited a graft survival of at least five years.

Excimer-PKP was performed in 74 eyes of 51 patients (GCD: *n* = 27/LCD: *n* = 21/MCD: *n* = 26). Of these, 61 eyes underwent primary Excimer-PKP (GCD: *n* = 19/LCD: *n* = 17/MCD: *n* = 25) and 13 eyes repeat Excimer-PKP (GCD: *n* = 8/LCD: *n* = 4/MCD: *n* = 1). The trephination size of Excimer-PKP was 7.0/7.1 mm in 5 eyes (LCD: *n* = 1/MCD: *n* = 4), 8.0/8.1 mm in 63 eyes (GCD: *n* = 25/LCD: *n* = 17/MCD: *n* = 21) and 8.5/8.6 mm in 6 eyes (GCD: *n* = 2/LCD: *n* = 3/MCD: *n* = 1).

The time from primary PKP to repeat PKP was 17.6 ± 7.2 years for GCD (range: 5.1–29.5 years), 21.5 ± 5.2 years for LCD (range: 13.3–26.3 years), and 39 years for MCD.

The follow-up of the entire study population was 7.5 ± 7.8 years (range: 0.4–40.1 years) for GCD, 8.4 ± 7.9 (range: 0.7–30.8 years) for LCD and 6.8 ± 9.2 (range: 0.5–37.9 years) for MCD. Follow-up at 5y + was 12.7 ± 8. 5 years (range: 6.1–40.1 years) for GCD, 14.4 ± 7.2 years (range: 5.4–30.8 years) for LCD and 16.3 ± 11.8 years (range: 6.1–37.9 years) for MCD.

Follow-up with tomographic measurements (number of eyes: GCD/LCD/MCD) were performed, after 6w-6 m (20/17/25), 1y (24/17/24), 1 st suture out (20/16/21), 2nd suture out (21/14/19), 3y (12/10/14), 4y (9/4/8), 5y (6/4/6) and 5y+ (23/17/8).

**Fig. 1:**
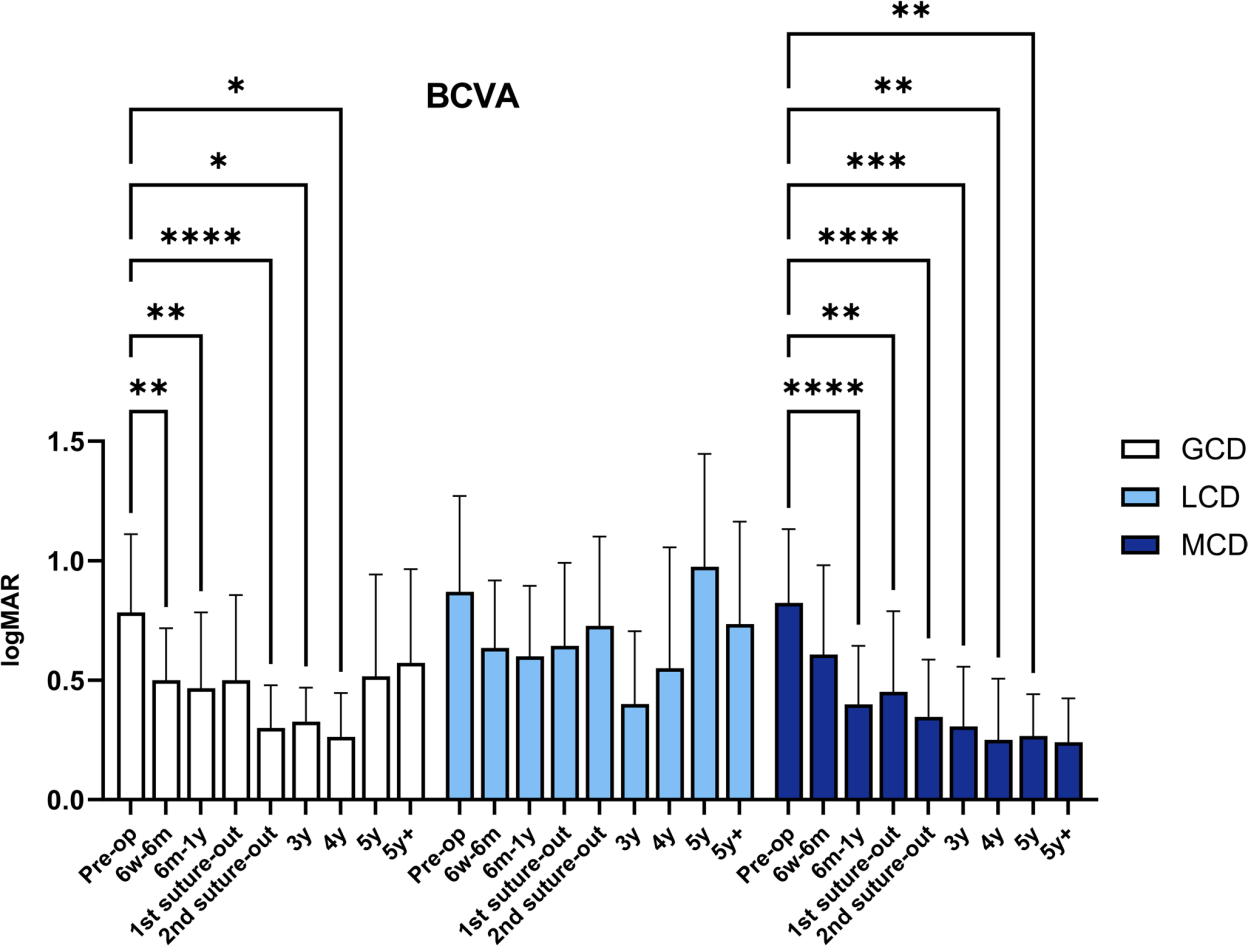
Visual outcome (in logMAR, logarithm of the minimum angle of resolution) in eyes with granular corneal dystrophy (GCD), lattice corneal dystrophy (LCD), and macular corneal dystrophy (MCD) after penetrating keratoplasty. Visual results (mean ± standard deviation) are presented preoperatively and postoperatively after 6 weeks to 6 months (6w-6 m), 1 year (y), after 1 st and 2nd suture removal, and then annually up to more than 5 years (5y+). Postoperative values were compared with preoperative values within the same group. Significant values are highlighted with asterisks (* *p* ≤ 0.05/** *p* ≤ 0.01/*** *p* ≤ 0.001/**** *p* ≤ 0.0001)

**Fig. 2:**
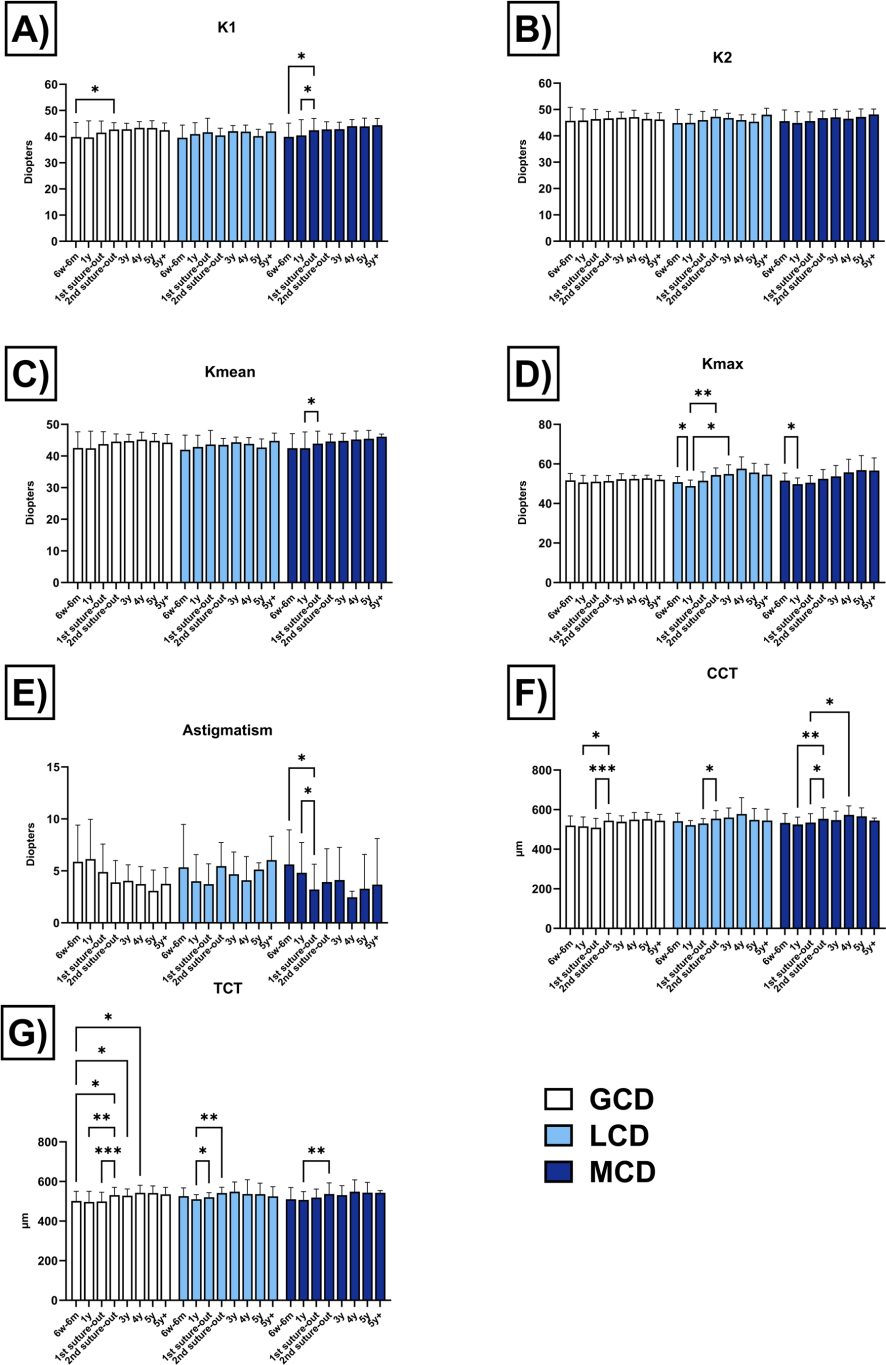
Corneal tomographic parameters of the anterior corneal surface and corneal thickness measurements in eyes with granular corneal dystrophy (GCD), lattice corneal dystrophy (LCD), and macular corneal dystrophy (MCD) after penetrating keratoplasty. The following parameters (in Diopters, D) were assessed: keratometry of the flat (K1, Fig. 2**A**) and steep (K2, Fig. 2**B**) meridian, mean keratometry (Kmean, Fig. 2**C**), maximum keratometry (Kmax, Fig. 2**D**), and astigmatism (Fig. 2**E**). Corneal pachymetry (in µm) included the central corneal thickness (CCT, Fig. 2**F**) and the thinnest corneal thickness (TCT, Fig. 2**G**). Tomographic parameters (mean ± standard deviation) are presented postoperatively after 6 weeks to 6 months (6w-6 m), 1 year (y), after 1 st and 2nd suture removal, and then annually up to more than 5 years (5y+). Values were compared within the same group. Significant values are highlighted with asterisks (* *p* ≤ 0.05/** *p* ≤ 0.01/*** *p* ≤ 0.001)

Visual outcomes following PKP are presented in Fig. 1; Table 1. A significant postoperative improvement in BCVA was observed in eyes with GCD and MCD, with sustained visual gains up to 4y and 5y postoperatively, respectively. Although a trend towards improved BCVA was also observed in eyes with LCD, these changes did not reach statistical significance. Tomographic parameters including K1, K2, Kmean, Kmax, astigmatism, CCT, and TCT are shown in Fig. 2 and summarized in Table 2. The endothelial cell density over time is provided in the Supplementary Information 1.

**Table 1:** Visual outcome (in logMAR, logarithm of the minimum angle of Resolution) in eyes with granular corneal dystrophy (GCD), lattice corneal dystrophy (LCD) and macular corneal dystrophy (MCD) after penetrating keratoplasty. The visual results (mean ± standard deviation) are shown preoperatively (Pre-op) and postoperatively after 6 weeks to 6 months (6w-6 m), 1 year (y), after 1 st and 2nd suture removal, and then annually up to more than 5 years (5y+). The square bracket contains the range. The preoperative baseline values were compared with the postoperative values within a group. Significant values are highlighted with asterisks (* *p* ≤ 0.05/** *p* ≤ 0.01/*** *p* ≤ 0.001/**** *p* ≤ 0.0001)

	Pre-op	6w-6 m	1y	1 st suture out	2nd suture out	3y	4y	5y	5y+
GCD	0.8 ± 0.3 [0.4–1.4] (*n* = 25)	0.5 ± 0.2 [0.2–1.0.2.0] (*n* = 20) **	0.5 ± 0.3 [0.1–1.3] (*n* = 24) **	0.5 ± 0.4 [0.1–1.4] (*n* = 20)	0.3 ± 0.2 [0.0–0.6.0.6] (*n* = 21) ****	0.3 ± 0.1 [0.0–0.5.0.5] (*n* = 11) *	0.3 ± 0.2 [0.1–0.6] (*n* = 8) *	0.5 ± 0.4 [0.1–1.3] (*n* = 6)	0.6 ± 0.4 [0.2–1.3] (*n* = 22)
LCD	0.9 ± 0.4 [0.4–1.3] (*n* = 17)	0.6 ± 0.3 [0.2–1.3] (*n* = 17)	0.6 ± 0.3 [0.1–1.3] (*n* = 18)	0.6 ± 0.3 [0.2–1.4] (*n* = 16)	0.7 ± 0.4 [0.1–1.4] (*n* = 14)	0.4 ± 0.3 [0.1–1.0.1.0] (*n* = 10)	0.6 ± 0.5 [0.2–1.3] (*n* = 4)	1.0 ± 0.5 [0.3–1.3] (*n* = 4)	0.7 ± 0.4 [0.1–1.4] (*n* = 17)
MCD	0.8 0.3 [0.3–1.3] (*n* = 21)	0.6 ± 0.4 [0.1–1.3] (*n* = 25)	0.4 ± 0.2 [0.1–1.0.1.0] (*n* = 24) ****	0.5 ± 0.3 [0.1–1.3] (*n* = 21) **	0.3 ± 0.2 [0.1–1.0.1.0] (*n* = 19) ****	0.3 ± 0.2 [0.1–0.8] (*n* = 14) ***	0.3 ± 0.3 [0.0–0.7.0.7] (*n* = 8) **	0.3 ± 0.2 [0.0–0.5.0.5] (*n* = 6) **	0.2 ± 0.2 [0.0–0.6.0.6] (*n* = 10)

**Table 2:** Corneal tomographic parameters of the anterior corneal surface and corneal thickness measurements in eyes with granular corneal dystrophy (GCD), lattice corneal dystrophy (LCD) and macular corneal dystrophy (MCD) after penetrating keratoplasty. The following parameters (in Diopters, D) were assessed: keratometry of the flat (K1) and steep (K2) meridian, mean keratometry (Kmean), maximum keratometry (Kmax) and astigmatism. Corneal pachymetry (in µm) included the central corneal thickness (CCT) and the thinnest corneal thickness (TCT). Tomographic parameters (mean ± standard deviation) are presented postoperatively after 6 weeks to 6 months (6w-6 m), 1 year (y), after 1 st and 2nd suture removal, and then annually up to more than 5 years (5y+)

		6w-6 m	1y	1 st suture out	2nd suture out	3y	4y	5y	5y+
K1 (D)	**GCD**	39.9 ± 5.6	39.7 ± 6.3	41.5 ± 4.5	42.7 ± 2.6	42.8 ± 2.3	43.4 ± 2.3	43.3 ± 2.7	42.5 ± 2.8
	**LCD**	39.6 ± 4.9	41.0 ± 4.4	41.7 ± 5.4	40.5 ± 2.7	42.1 ± 2.1	41.9 ± 2.5	40.2 ± 2.6	42.0 ± 2.9
	**MCD**	39.9 ± 5.2	40.5 ± 6.0	42.4 ± 4.5	42.8 ± 2.9	42.9 ± 2.6	44.0 ± 2.5	43.9 ± 3.2	44.4 ± 2.6
K2 (D)	**GCD**	45.7 ± 5.1	45.8 ± 4.4	46.4 ± 3.6	46.6 ± 2.7	46.9 ± 2.2	47.1 ± 2.6	46.4 ± 2.2	46.2 ± 2.5
	**LCD**	44.9 ± 5.1	45.0 ± 3.2	46.0 ± 3.2	47.2 ± 2.7	46.8 ± 1.8	46.0 ± 1.9	45.4 ± 2.8	48.0 ± 2.4
	**MCD**	45.6 ± 4.3	44.9 ± 4.2	45.6 ± 3.5	46.7 ± 2.7	47.0 ± 3.1	46.5 ± 2.8	47.2 ± 3.1	48.1 ± 2.1
Kmean (D)	**GCD**	42.5 ± 5.1	42.5 ± 5.4	43.8 ± 3.9	44.5 ± 2.4	44.7 ± 2.1	45.2 ± 2.3	44.8 ± 2.3	44.2 ± 2.6
	**LCD**	42.0 ± 4.6	42.8 ± 3.7	43.7 ± 4.5	43.5 ± 2.0	44.3 ± 1.6	43.9 ± 1.9	42.7 ± 2.7	44.8 ± 2.4
	**MCD**	42.5 ± 4.6	42.5 ± 5.1	43.9 ± 3.9	44.6 ± 2.3	44.8 ± 2.4	45.2 ± 2.6	45.5 ± 2.7	46.1 ± 0.8
Kmax (D)	**GCD**	51.7 ± 3.5	50.6 ± 3.6	51.0 ± 3.2	51.4 ± 2.8	52.2 ± 2.8	52.4 ± 1.8	52.7 ± 1.6	52.0 ± 2.1
	**LCD**	50.8 ± 2.8	48.8 ± 3.0	51.5 ± 4.4	54.4 ± 3.6	54.9 ± 4.6	57.6 ± 6.0	55.6 ± 4.7	54.6 ± 5.2
	**MCD**	51.6 ± 3.8	49.8 ± 3.2	50.5 ± 3.7	52.5 ± 4.7	53.7 ± 5.6	55.8 ± 6.6	56.9 ± 7.4	56.6 ± 6.4
Astigmatism (D)	**GCD**	5.9 ± 3.5	6.1 ± 3.8	4.9 ± 2.7	3.9 ± 2.1	4.0 ± 1.5	3.7 ± 1.7	3.1 ± 2.0	3.8 ± 1.6
	**LCD**	5.3 ± 4.1	4.0 ± 2.6	3.7 ± 2.0	5.5 ± 2.3	4.7 ± 2.1	4.1 ± 2.3	5.1 ± 0.6	6.0 ± 2.3
	**MCD**	5.6 ± 3.3	4.8 ± 2.9	3.2 ± 2.4	3.9 ± 3.2	4.1 ± 3.1	2.5 ± 0.6	3.3 ± 3.3	3.7 ± 4.4
CCT (µm)	**GCD**	519.4 ± 49.1	515.6 ± 47.3	508.9 ± 47.1	544.0 ± 36.7	538.8 ± 30.3	549.9 ± 35.8	552.2 ± 33.4	544.4 ± 31.2
	**LCD**	541.9 ± 40.4	521.6 ± 23.3	530.6 ± 24.1	554.5 ± 40.8	560.4 ± 48.1	578.3 ± 82.1	548.0 ± 57.9	545.0 ± 57.3
	**MCD**	532.8 ± 47.6	525.0 ± 37.6	534.9 ± 44.7	553.3 ± 56.2	547.0 ± 45.5	573.7 ± 45.0	566.2 ± 43.1	545.3 ± 12.1
TCT (µm)	**GCD**	501.7 ± 48.5	497.0 ± 53.3	499.3 ± 46.1	531.1 ± 39.5	528.4 ± 33.6	543.1 ± 38.0	541.4 ± 36.3	535.2 ± 35.0
	**LCD**	526.4 ± 41.3	510.1 ± 23.3	520.1 ± 23.7	541.5 ± 29.7	548.6 ± 48.8	536.8 ± 72.5	536.3 ± 55.2	525.3 ± 48.6
	**MCD**	510.3 ± 59.2	507.0 ± 41.7	519.1 ± 42.5	536.8 ± 56.0	531.0 ± 47.8	548.4 ± 60.2	543.3 ± 52.4	542.8 ± 11.9

### Corneal densitometry

**Fig. 3:**
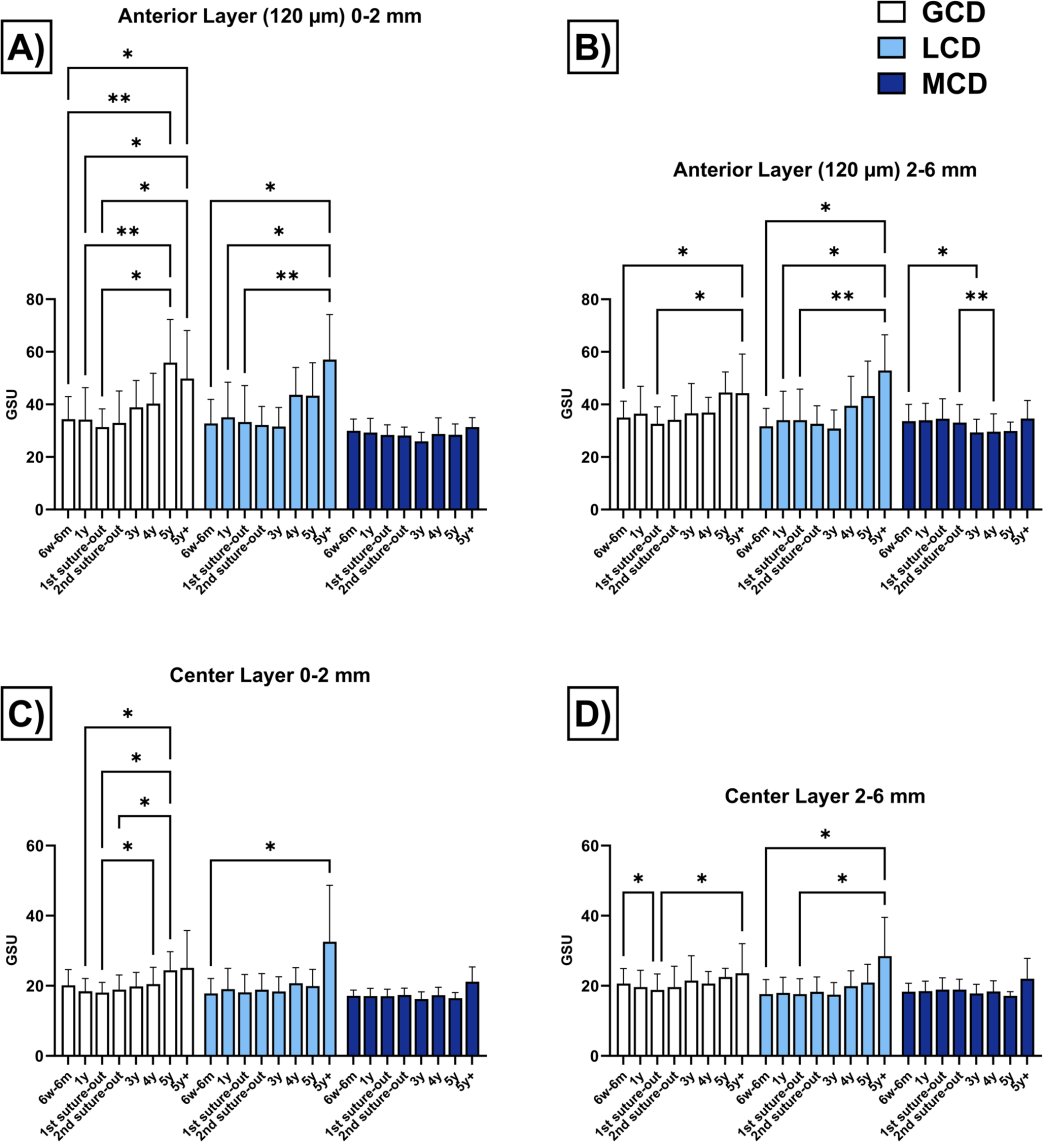
Corneal densitometry measurements (in grayscale units, GSU) of the anterior corneal layer (anterior 120 μm, Fig. 3**A** and **B**) and the center corneal layer (volume between the anterior 120 μm and posterior 60 μm, Fig. 3**C** and **D**) in eyes with granular corneal dystrophy (GCD), lattice corneal dystrophy (LCD), and macular corneal dystrophy (MCD) after penetrating keratoplasty. The measurements (mean ± standard deviation) of different diameters (0–2 and 2–6 mm) are presented postoperatively after 6 weeks to 6 months (6w-6 m), 1 year (y), after 1 st and 2nd suture removal, and then annually up to more than 5 years (5y+). Values were compared within the same group. Significant values are highlighted with asterisks (* *p* ≤ 0.05/** *p* ≤ 0.01)

**Fig. 4:**
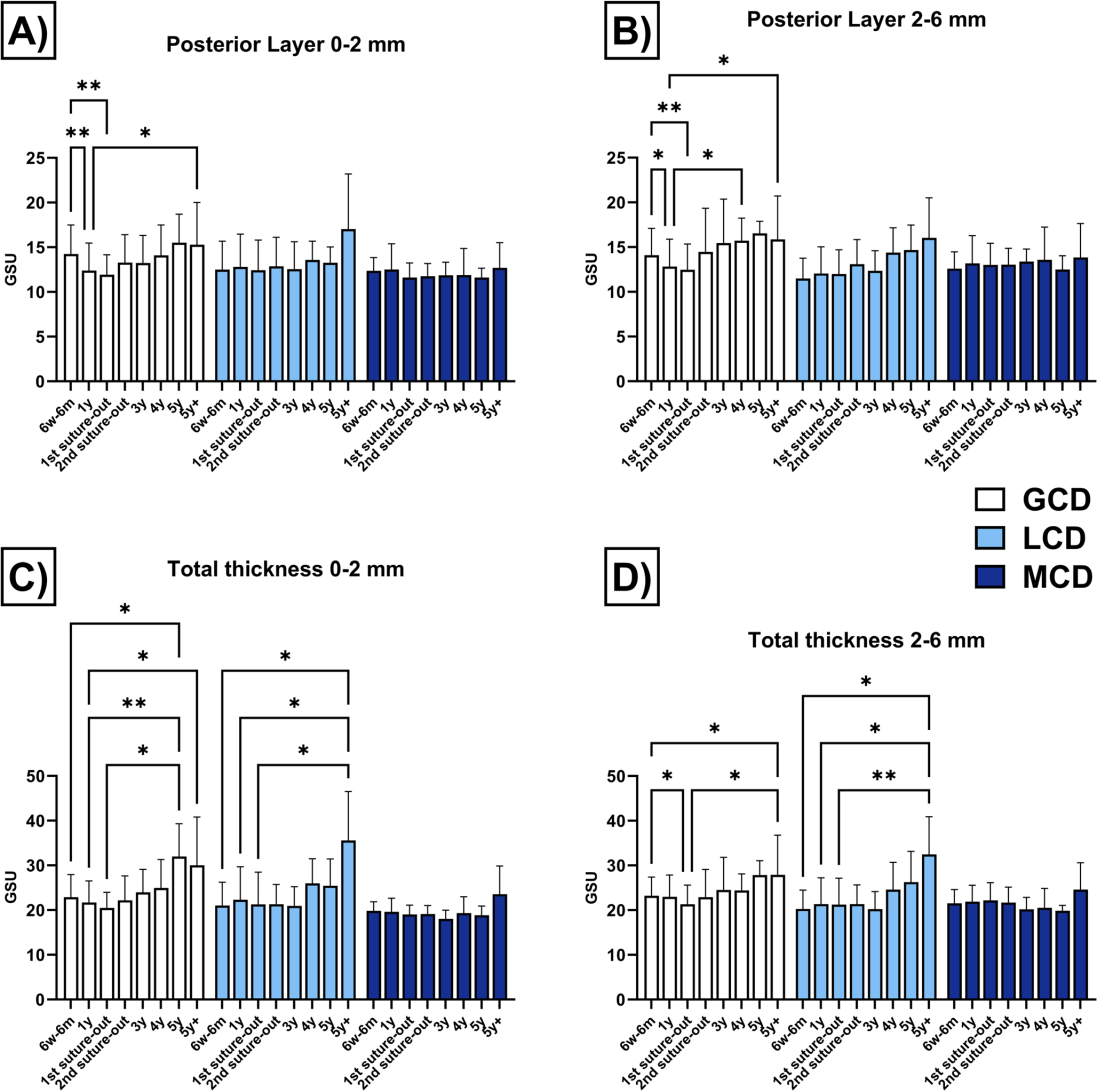
Corneal densitometry measurements (in grayscale units, GSU) of the posterior corneal layer (posterior 60 μm, Fig. 4**A** and **B**) and the total corneal thickness (Fig. 4**C** and **D**) in eyes with granular corneal dystrophy (GCD), lattice corneal dystrophy (LCD), and macular corneal dystrophy (MCD) after penetrating keratoplasty. The measurements (mean ± standard deviation) of different diameters (0–2 and 2–6 mm) are presented postoperatively after 6 weeks to 6 months (6w-6 m), 1 year (y), after 1 st and 2nd suture removal, and then annually up to more than 5 years (5y+). Values were compared within the same group. Significant values are highlighted with asterisks (* *p* ≤ 0.05/** *p* ≤ 0.01)

The densitometry values are shown in Table 3 and are graphically represented in Fig. 3 (anterior and central corneal layer) and Fig. 4 (posterior corneal layer and total corneal thickness).

**Table 3:** Corneal densitometry (in grayscale units) for the anterior corneal layer (120 μm), the central corneal layer (between the anterior 120 μm and posterior 60 μm), and the total corneal thickness in eyes with granular corneal dystrophy (GCD), lattice corneal dystrophy (LCD) and macular corneal dystrophy (MCD) following penetrating keratoplasty

			6w-6 m	1y	1 st suture out	2nd suture out	3y	4y	5y	5y+
Anterior layer (120 μm)	**0–2 mm**	**GCD**	34.3 ± 8.6	34.2 ± 2.1	31.3 ± 6.8	32.9 ± 12.1	38.8 ± 10.2	40.2 ± 11.5	55.9 ± 16.4	49.8 ± 18.2
		**LCD**	32.7 ± 9.1	35.1 ± 13.3	33.3 ± 13.8	32.1 ± 7.0	31.5 ± 7.2	43.6 ± 10.4	43.3 ± 12.4	57.0 ± 17.0
		**MCD**	29.9 ± 4.4	29.2 ± 5.3	28.3 ± 3.8	28.1 ± 3.1	25.9 ± 3.3	28.7 ± 6.1	28.4 ± 4.1	31.3 ± 3.6
	**2–6 mm**	**GCD**	35.0 ± 6.2	36.5 ± 10.4	32.6 ± 6.5	34.2 ± 9.1	36.7 ± 11.3	36.9 ± 5.9	44.6 ± 7.8	44.3 ± 14.9
		**LCD**	31.7 ± 6.8	34.0 ± 1.0	34.0 ± 1.8	32.6 ± 6.8	30.8 ± 7.1	39.5 ± 1.2	43.2 ± 3.3	52.9 ± 3.6
		**MCD**	33.6 ± 6.4	34.0 ± 6.5	34.6 ± 7.6	33.1 ± 6.8	29.3 ± 5.0	29.7 ± 6.8	29.9 ± 3.4	34.7 ± 6.8
Center layer	**0–2 mm**	**GCD**	20.1 ± 4.5	18.4 ± 3.7	18.1 ± 2.9	18.9 ± 4.2	19.8 ± 4.0	20.5 ± 4.8	24.5 ± 5.3	25.1 ± 10.7
		**LCD**	17.8 ± 4.3	19.0 ± 5.9	18.1 ± 5.1	18.9 ± 4.6	18.4 ± 4.2	20.7 ± 4.4	19.9 ± 4.7	32.6 ± 16.1
		**MCD**	17.1 ± 1.6	17.1 ± 2.2	17.0 ± 2.0	17.4 ± 2.0	16.2 ± 2.0	17.3 ± 2.2	16.5 ± 1.6	21.2 ± 4.2
	**2–6 mm**	**GCD**	20.6 ± 4.3	19.6 ± 4.8	18.8 ± 4.6	19.6 ± 6.0	21.5 ± 7.1	20.6 ± 3.4	22.5 ± 2.5	23.6 ± 8.4
		**LCD**	17.6 ± 4.1	18.0 ± 4.5	17.7 ± 4.4	18.3 ± 4.2	17.5 ± 3.4	19.9 ± 4.4	21.0 ± 5.2	28.5 ± 11.0
		**MCD**	18.3 ± 2.4	18.5 ± 2.9	18.9 ± 3.3	18.9 ± 2.9	17.8 ± 2.6	18.4 ± 3.1	17.1 ± 1.2	22.0 ± 5.8
Posterior layer	**0–2 mm**	**GCD**	14.2 ± 3.3	12.4 ± 3.1	11.9 ± 2.2	13.3 ± 3.1	13.2 ± 3.1	14.1 ± 3.4	15.5 ± 3.2	15.3 ± 4.7
		**LCD**	12.5 ± 3.2	12.8 ± 3.7	12.4 ± 3.4	12.9 ± 3.2	12.6 ± 3.1	13.6 ± 2.1	13.3 ± 1.8	17.0 ± 6.2
		**MCD**	12.4 ± 1.5	12.5 ± 2.9	11.6 ± 1.6	11.8 ± 1.4	11.9 ± 1.4	11.9 ± 3.0	11.6 ± 1.0	12.7 ± 2.8
	**2–6 mm**	**GCD**	14.1 ± 3.0	12.8 ± 3.0	12.5 ± 2.9	14.5 ± 4.9	15.5 ± 4.9	15.7 ± 2.5	16.5 ± 1.4	15.9 ± 4.8
		**LCD**	11.5 ± 2.3	12.0 ± 3.0	12.0 ± 2.7	13.1 ± 2.7	12.3 ± 2.3	14.4 ± 2.8	14.7 ± 2.8	16.0 ± 4.5
		**MCD**	12.6 ± 1.9	13.2 ± 3.1	13.0 ± 2.4	13.0 ± 1.8	13.4 ± 1.4	13.6 ± 3.7	12.5 ± 1.5	13.9 ± 3.8
Complete corneal thickness	**0–2 mm**	**GCD**	22.9 ± 5.0	21.7 ± 4.8	20.5 ± 3.5	22.2 ± 5.5	24.0 ± 5.1	25.0 ± 6.4	32.0 ± 7.4	30.0 ± 10.8
		**LCD**	21.0 ± 5.2	22.3 ± 7.4	21.3 ± 7.2	21.3 ± 4.4	20.9 ± 4.3	26.0 ± 5.5	25.5 ± 6.0	35.6 ± 11.0
		**MCD**	19.8 ± 2.0	19.6 ± 3.0	19.0 ± 2.1	19.1 ± 1.9	18.0 ± 2.0	19.3 ± 3.7	18.9 ± 2.1	23.5 ± 6.3
	**2–6 mm**	**GCD**	23.2 ± 4.2	23.0 ± 4.9	21.3 ± 4.3	22.9 ± 6.2	24.5 ± 7.3	24.4 ± 3.7	27.9 ± 3.2	27.9 ± 8.9
		**LCD**	20.3 ± 4.2	21.4 ± 5.9	21.2 ± 5.9	21.3 ± 4.3	20.2 ± 4.0	24.6 ± 6.1	26.3 ± 6.9	32.5 ± 8.4
		**MCD**	21.5 ± 3.1	21.9 ± 3.7	22.2 ± 3.9	21.7 ± 3.5	20.2 ± 2.7	20.5 ± 4.3	19.8 ± 1.2	24.6 ± 6.0

Postoperative values (mean ± standard deviation) are displayed for different corneal diameters (0–6 mm) after 6 weeks to 6 months (6w-6 m), 1 year (y), after 1 st and 2nd suture removal, and then annually up to more than 5 years (5y+).

#### Anterior Layer (0–2 mm)

In eyes with GCD, GSU values were higher at 5y postoperatively compared to values after 6w-1 m (*p* = 0.0087), 1y (*p* = 0.0040) and 1 st suture out (*p* = 0.0254). At 5y+, GSU values remained elevated compared to the same earlier time points (*p* = 0.0283/*p* = 0.0396/*p* = 0.0489). For LCD, GSU values were increased after 5y + compared to 6w-6 m (*p* = 0.0192), 1y (*p* = 0.0160) and 1 st suture out (*p* = 0.0071). In contrast, eyes with MCD showed no changes in GSU values throughout the entire observation period.

#### Anterior Layer (2–6 mm)

In eyes with GCD, GSU values were higher at 5y + compared to values recorded at 6w-6 m (*p* = 0.0285) and 1 st suture out (*p* = 0.0397). LCD eyes showed elevated GSU values after 5y + when compared to 6w-6 m (*p* = 0.0265), 1y (*p* = 0.0166), and 1 st suture out (*p* = 0.0064). In contrast, eyes with MCD exhibited a decrease in GSU values at 3y compared to 6w-6 m (*p* = 0.0399), and values remained lower at 4y when compared to those measured at 2nd suture out (*p* = 0.0048).

#### Central Layer (0–2 mm)

In GCD eyes, GSU values were increased at 5y postoperatively compared to 1y (*p* = 0.0369), 1 st suture out (*p* = 0.0403) and 2nd suture out (*p* = 0.0330). Additionally, values at 4y were elevated compared to 1 st suture out (*p* = 0.0498). In LCD eyes, GSU values were increased at 5y + compared to 6w-6 m (*p* = 0.0279), while MCD eyes showed no postoperative changes in GSU values.

#### Central Layer (2–6 mm)

For GCD, GSU values were initially lower at 1 st suture out compared to 6w-6 m (*p* = 0.0298), but were higher at 5y + than at 1 st suture out (*p* = 0.0382). LCD eyes exhibited elevated GSU values after 5y + compared to 6w-6 m (*p* = 0.0460) and 1 st suture out (*p* = 0.0250). No changes were observed in MCD eyes throughout the follow-up period.

#### Posterior Region (0–2 mm)

In GCD, GSU values decreased at 1y (*p* = 0.0014) and 1 st suture out (*p* = 0.0055) compared to values at 6w-6 m. However, at 5y+, GSU values were increased compared to 1y (*p* = 0.0451). In contrast, no changes in GSU values were observed in LCD or MCD.

#### Posterior Region (2–6 mm)

In GCD eyes, GSU values were lower at 1y (*p* = 0.0110) and at 1 st suture out (*p* = 0.0021) compared to 6w-6 m. Increased GSU values were observed at 4y (*p* = 0.0430) and 5y+ (*p* = 0.0299) compared to 1y. No changes were detected for LCD or MCD.

#### Total Corneal Thickness (0–2 mm)

For GCD, GSU values were increased at 5y compared to 6w-6 m (*p* = 0.0178), 1y (*p* = 0.0067), and 1 st suture out (*p* = 0.0203). Additionally, values remained elevated at 5y + when compared to 1y (*p* = 0.0474). LCD eyes showed higher GSU values after 5y + compared to 6w-6 m (*p* = 0.0186), 1y (*p* = 0.0463), and 1 st suture out (*p* = 0.0229). No differences in GSU values were observed in MCD.

#### Total Corneal Thickness (2–6 mm)

In GCD eyes, GSU values were lower at 1 st suture out than at 6w-6 m (*p* = 0.0394), but increased at 5y+ (*p* = 0.0441) when compared to 6w-6 m (p=) and 1 st suture out (*p* = 0.0419). For LCD, increased GSU values were recorded after 5y + compared to 6w-6 m (*p* = 0.0365), 1y (*p* = 0.0329) and at 1 st suture out (*p* = 0.0091). In contrast, no changes were observed in MCD eyes.

### Linear regression analysis

**Fig. 5:**
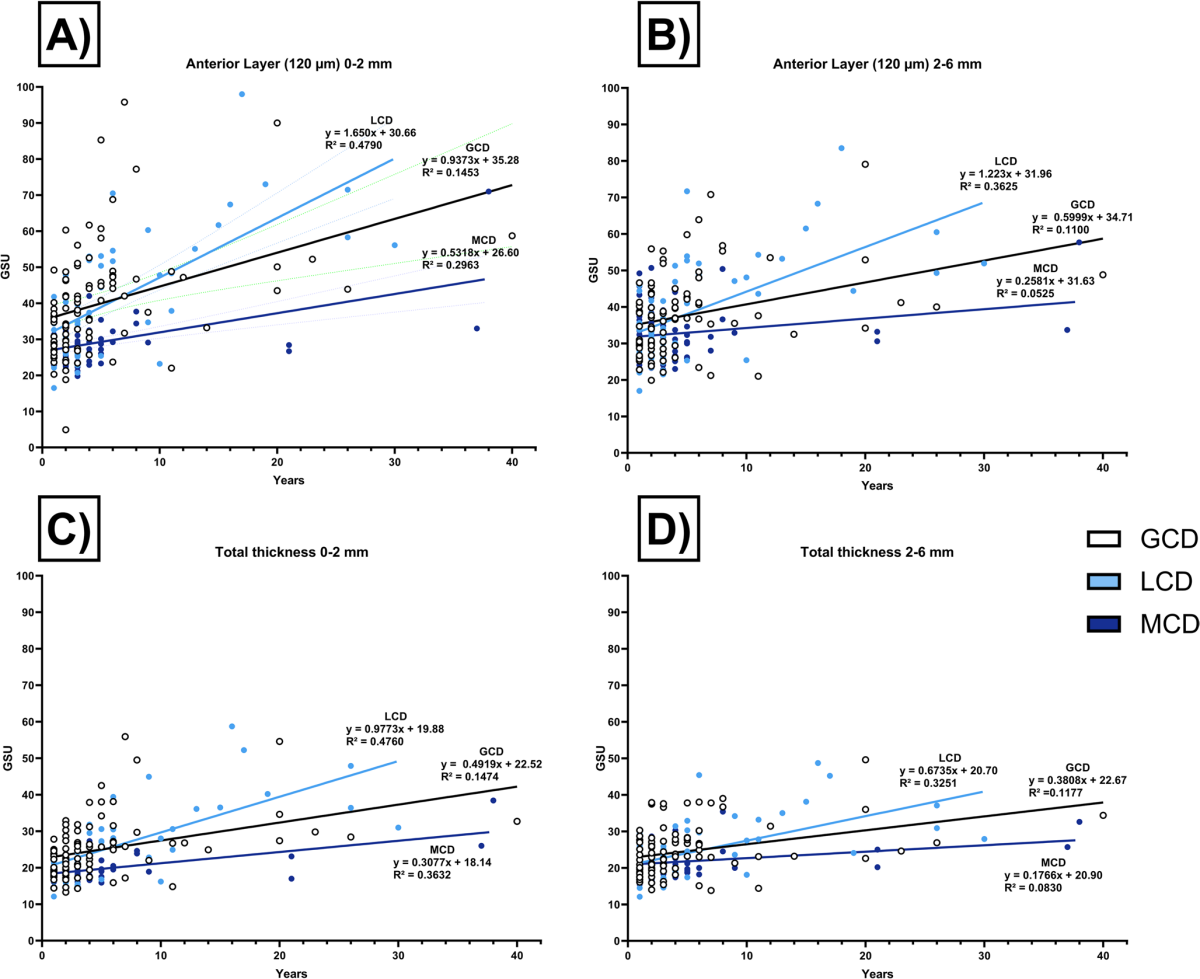
Longitudinal assessment of corneal densitometry in eyes with granular corneal dystrophy (GCD), macular corneal dystrophy (MCD) and lattice corneal dystrophy (LCD) after penetrating keratoplasty. Scatter plots with regression lines (Figs. 5**A**–**D**) show grayscale unit (GSU) values over time (in years) for each dystrophy across different corneal regions: anterior layer 0–2 mm (Fig. 5**A**), anterior layer 2–6 mm (Fig. 5**B**), total corneal thickness 0–2 mm (Fig. 5**C**) and total corneal thickness 2–6 mm (Fig. 5**D**). Linear regression equations and corresponding R² values indicate the rate and strength of GSU progression over time. LCD and GCD show a steeper increase in GSU values over time, while MCD demonstrates minimal change across all regions

In the anterior layer (120 μm, 0–2 mm) (Fig. 5A), LCD and GCD showed increased GSU values over time, with regression slopes of 1.650 (R²=0.4790) and 0.9373 (R²=0.1453), respectively. MCD demonstrated a weaker trend with a slope of 0.5316 and lower explanatory power (R²=0.2953). Also, in the 2–6 mm zone of the anterior layer (120 μm) (Fig. 5B), LCD again exhibited the steepest increase in backscatter over time (slope = 1.223, R²=0.3625), followed by GCD (slope = 0.5999, R²=0.1100), while MCD showed minimal progression (slope = 0.2581, R²=0.0525).

Analysis of the densitometry of the total corneal thickness (0–2 mm) (Fig. 5C) revealed a similar pattern. LCD showed the greatest increase in GSU over time (slope = 0.9773, R²=0.4760), with GCD following (slope = 0.4919, R²=0.1474), and MCD again showing minimal change (slope = 0.3077, R²=0.3632). In the total corneal thickness (2–6 mm) (Fig. 5D), the most prominent changes were again observed in LCD (slope = 0.6735, R²=0.3251) and GCD (slope = 0.3808, R² = 0.1177), while MCD showed a low increase in GSU (slope = 0.1766, R²=0.0830).

### Clinical evaluation of recurrences in the graft

**Fig. 6:**
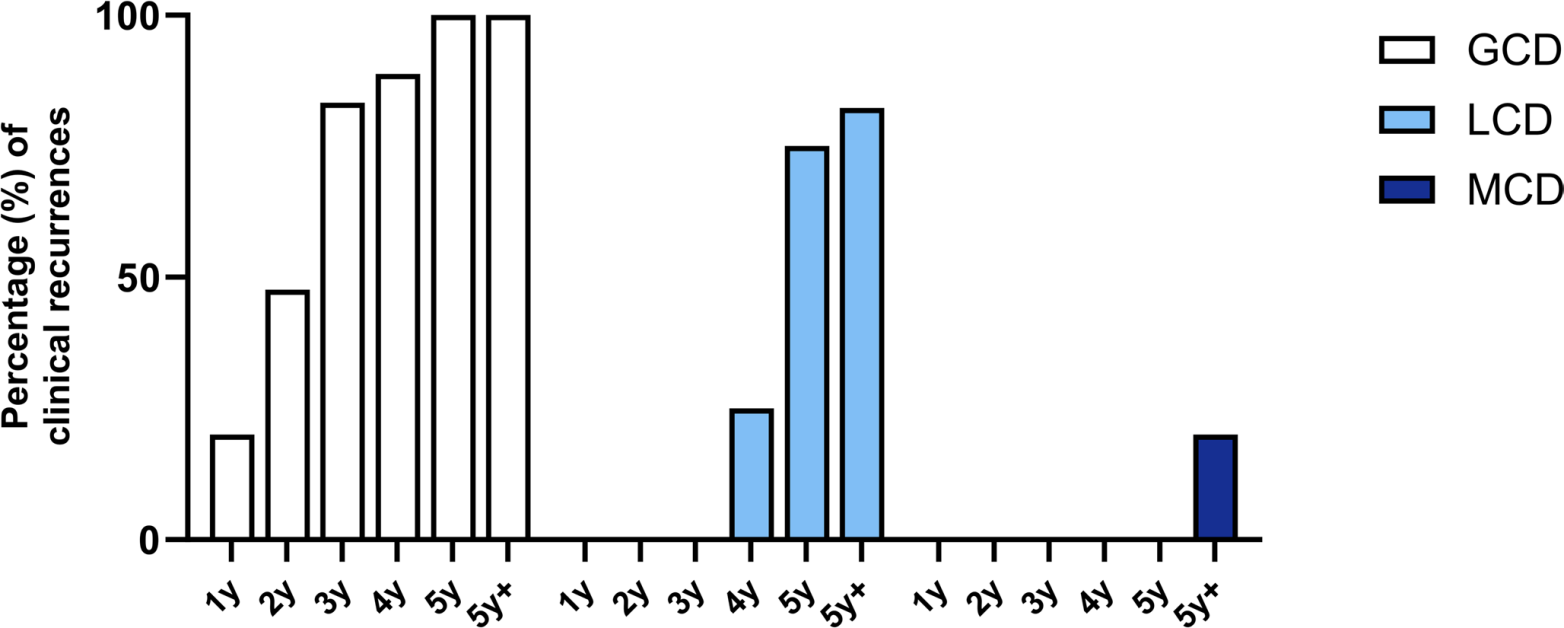
Cumulative recurrence rate in eyes with granular corneal dystrophy (GCD), macular corneal dystrophy (MCD) and lattice corneal dystrophy (LCD) after penetrating keratoplasty at defined postoperative intervals (1, 2, 3, 4, 5, and > 5 years). GCD demonstrated the earliest onset and highest cumulative recurrence rate, with all eyes affected by year 5. LCD showed a later onset, with recurrences first observed at year 4. In contrast, MCD was characterized by rare, isolated recurrences, which appeared only beyond 5 years postoperatively

The temporal distribution of clinical recurrences across the different corneal dystrophies is illustrated in Fig. 6. Recurrence was defined as the appearance of any morphological changes of the graft indicative of the underlying dystrophy, without clinical differentiation between mild and severe recurrences. No signs of recurrence were observed within the first six months postoperatively in any of the dystrophies (GCD, LCD, or MCD). The earliest clinical recurrences appeared in GCD after 1y, followed by LCD at 4y and MCD at 5y + postoperatively. In GCD, recurrences initially manifested as fine, centrally located, superficial opacities within the previously clear graft, which progressively increased in density and depth over time. By 2y postoperatively, nearly 50% of GCD eyes exhibited superficial graft recurrences. Compared to LCD and MCD, GCD demonstrated a notably higher recurrence frequency, resulting in clinical recurrence in all grafts by 5y.

In contrast, LCD recurrences were first detected at 4y postoperatively, presenting as diffuse subepithelial and anterior stromal opacities. Notably, these changes were not accompanied by the characteristic lattice lines within the graft. Recurrences in MCD appeared as discrete stromal opacities in the peripheral graft.

## Discussion

The incidence of recurrence after PKP or DALK varies considerably depending on the underlying corneal dystrophy. More than 30 years ago, Lyons et al. reported early-onset recurrences of GCD, characterized by a superficial, vortex-like pattern in the central cornea, indicative of epithelial involvement [14]. At that time, however, GCD was still considered a stromal corneal dystrophy, and the first description of pathogenic mutations in the *TGFBI* gene were published in 1994 and 1997 [15, 16]. The second edition of the IC3D classification, published in 2015, was the first to formally introduce the category of epithelial-stromal *TGFBI* corneal dystrophies [17]. This represented a significant advancement in the pathophysiological understanding of these disorders, as it acknowledged not only the stromal but also the epithelial involvement, thereby providing a more comprehensive framework for their classification and clinical interpretation. In contrast to epithelial-stromal *TGFBI* corneal dystrophies, such as GCD or LCD, stromal dystrophies like MCD show a significantly lower tendency to recur and are associated with an overall more favorable long-term prognosis [2]. As the definition and classification of recurrences is not standardized in the existing literature, the present study investigated the recurrence rate after PKP in GCD, LCD and MCD with additional use of corneal densitometry measurement, as an objective and quantifiable measure. While corneal densitometry has been applied in various surgical settings such as Descemet membrane endothelial keratoplasty, Descemet stripping automated endothelial keratoplasty and DALK, it has not yet been used to assess the recurrence after PKP for GCD, LCD and MCD [18–21]. Therefore, the purpose of the present study was to provide a detailed long-term evaluation of graft clarity and recurrence after PKP combining clinical data with objective Scheimpflug-based corneal densitometry.

According to Lyons et al., superficial and centrally emphasized recurrences of GCD appear consistently within four years after keratoplasty, regardless of graft diameter or whether a lamellar or penetrating procedure was performed [14]. In contrast, Lewis et al. reported that significant recurrences are most delayed after PKP, with a mean interval of 13.7 years, compared to 3.7 years after anterior lamellar keratoplasty and 3.2 years after DALK [22]. Another study examining clinical recurrence rates of various corneal dystrophies following PKP found simple recurrences in 17.8% of eyes with LCD and 40.0% of eyes with GCD within a five-year follow-up period. Clinically significant recurrences, however, were observed in 17.1% of eyes with LCD, but in none of the GCD eyes. Notably, neither simple nor clinically significant recurrences were observed in MCD during the same follow-up interval [23].

Conversely, clinical recurrences in stromal dystrophies, particularly in MCD, are considered rare and typically of limited clinical significance as manifestations generally do not occur for several decades [24, 25]. Histologically, subepithelial glycosaminoglycan deposits were found at the graft margin without involvement of the optic center [26]. In addition, a recent meta-analysis has shown a significantly higher risk of recurrence in MCD eyes who have undergone DALK, with an increased incidence of interface opacities reported [27]. The present study confirms that GCD occurs earliest and has the highest rate of clinical recurrence, with all grafts showing signs of recurrence within five years, which is consistent with the findings reported by Lyons et al. [14]. In contrast, recurrence in LCD was more delayed but occurred at a higher frequency than previously reported in the literature. Notably, the subepithelial and anterior stromal opacities observed in LCD grafts may partially result from impaired epithelial adhesion, recurrent epithelial erosions, and secondary scarring [28].

Corneal densitometry proved to be a reliable indicator of progressive graft opacification, especially in GCD and LCD. A significant increase in light backscatter was observed especially in the anterior and central layers and was particularly pronounced after five years. Importantly, these densitometric changes often occurred in parallel with the appearance of clinically detectable recurrences. In contrast, MCD showed only minimal changes in densitometry values across all corneal regions and time points, indicating a more stable postoperative course with a lower risk of recurrence and supporting the general assumption that recurrences in stromal dystrophies are rare. The longitudinal assessment of corneal densitometry in this study provided a detailed evaluation of temporal changes in graft clarity across various corneal dystrophies. A key consideration is the marked interindividual variability, characterized by distinct morphological patterns and depths of stromal deposition. This variability, attributable to underlying phenotypic heterogeneity can significantly influence the onset, distribution, and progression of recurrence, and may impact the interpretation and comparability of densitometry data [29].

Overall, PKP offers favorable long-term visual outcomes in epithelial-stromal *TGFBI* and stromal corneal dystrophies, with the most stable results observed in MCD. In contrast, GCD and LCD are prone to early and progressive recurrence, particularly in the anterior cornea. Scheimpflug densitometry provides valuable, layer-specific insights into graft clarity and serves as an early marker for recurrence. Its integration into routine follow-up could significantly enhance postoperative care, enabling personalized monitoring and timely intervention in patients at higher risk.

## Supplementary Information

Below is the link to the electronic supplementary material.

Supplementary File 1 (DOCX 128 KB)
