# Supplementary material for: Objective assessment of graft clarity and recurrence after penetrating keratoplasty for granular, lattice and macular corneal dystrophy using scheimpflug densitometry
Source: Graefes Arch Clin Exp Ophthalmol. 2025 Dec 15;264(4):1033–45. doi: 10.1007/s00417-025-07050-x (PMC13002641; doi:10.1007/s00417-025-07050-x)
Supplement: Supplementary file 1 — (DOCX 128 KB) [file 417_2025_7050_MOESM1_ESM.docx]

**Supplementary Information**

**Article title:**

Objective assessment of graft clarity and recurrence after penetrating keratoplasty for Granular, Lattice and Macular Corneal Dystrophy using Scheimpflug densitometry

**Journal name:**

Graefe's Archive for Clinical and Experimental Ophthalmology

**Author names and affiliation:**

Tim Berger, MD, Berthold Seitz, MD, Elias Flockerzi, MD, Albéric Sneyers, MD, Shady Suffo, MD, Loay Daas, MD

Department of Ophthalmology, Saarland University Medical Center, Homburg/Saar, Germany

**Correspondence:**

Dr. Tim Berger, MD, FEBO

Department of Ophthalmology, Saarland University Medical Center, Kirrberger Str., D-66424 Homburg/Saar, Germany

Telephone number: 0049 6841 16-22302

Fax number: 0049 6841 16-22479

E-Mail: tim.berger@uks.eu


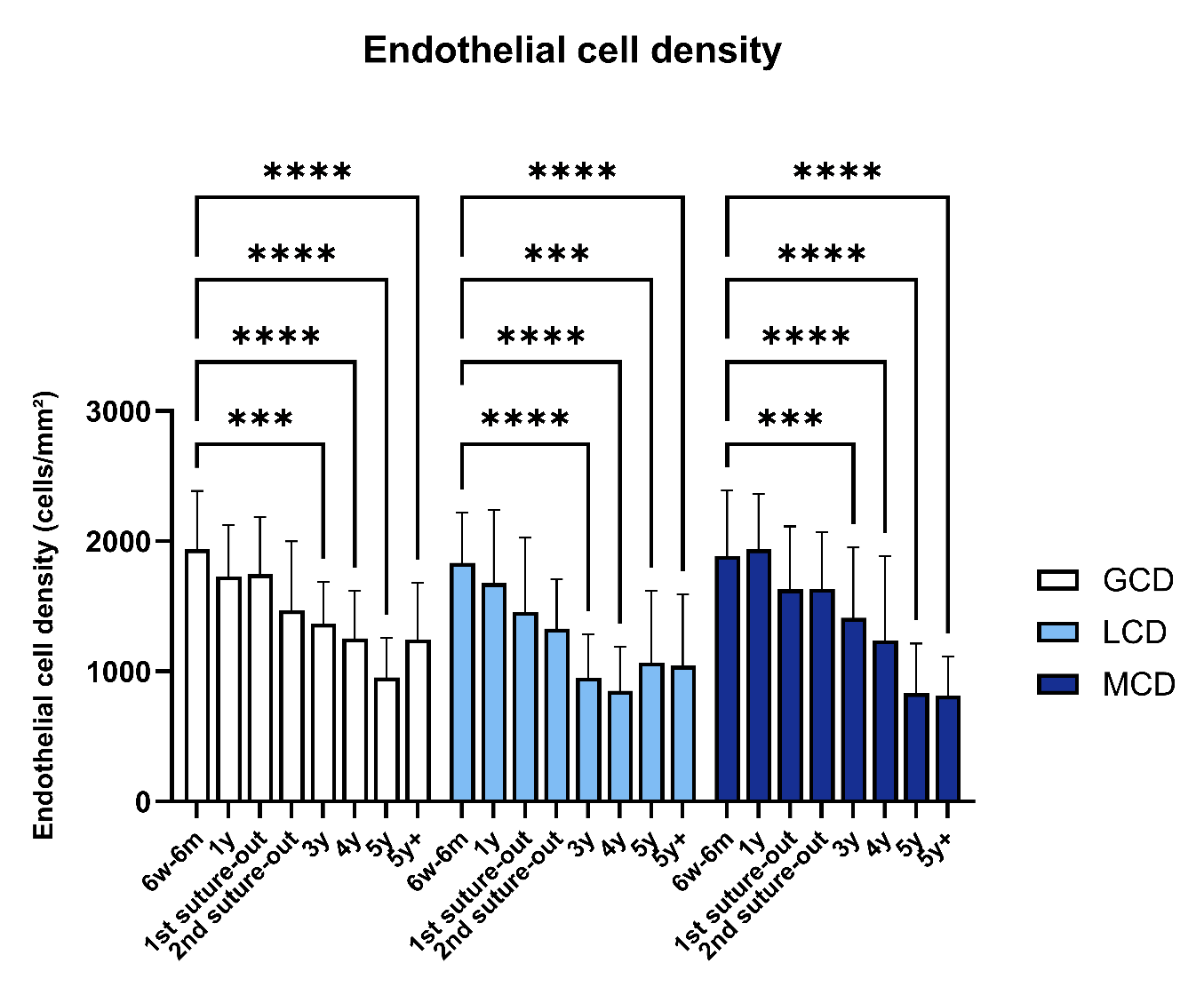
**Supplementary Figure 1:** Endothelial cell density (cells/mm², EM-3000, Tomey Corp., Nagoya, Japan) in eyes with granular corneal dystrophy (GCD), lattice corneal dystrophy (LCD), and macular corneal dystrophy (MCD) after penetrating keratoplasty. The measurements (mean ± standard deviation) are presented postoperatively after 6 weeks to 6 months (6w-6m), 1 year (y), after 1^st^ and 2^nd^ suture removal, and then annually up to more than 5 years (5y+). Values were compared within the same group. Significant values are highlighted with asterisks (*** p≤0.001/ **** p≤0.0001). Overall, a significant decrease in endothelial cell density is seen in all three groups.

|  | **6w-6m** | **1y** | **1^st^ suture out** | **2^nd^ suture out** | **3y** | **4y** | **5y** | **5y+** |
| --- | --- | --- | --- | --- | --- | --- | --- | --- |
| GCD | 1940 ± 447  (n=17) | 1729 ± 394  (n=22) | 1747 ± 437  (n=16) | 1469 ± 529  (n=18) | 1366 ± 323  (n=9) *** | 1253 ± 367  (n=7) **** | 953 ± 303  (n=3) **** | 1246 ± 432  (n=16) **** |
| LCD | 1834 ± 387  (n=15) | 1678 ± 559  (n=16) | 1457 ± 569  (n=13) | 1330 ± 379  (n=12) | 951 ± 333  (n=10) **** | 851 ± 340  (n=4) **** | 1069 ± 551  (n=4) *** | 1043 ± 548  (n=10) **** |
| MCD | 1885 ± 503  (n=23) | 1938 ± 423  (n=21) | 1632 ± 482  (n=22) | 1633 ± 439  (n=18) | 1411 ± 540  (n=14) *** | 1239 ± 645  (n=8) **** | 833 ± 380  (n=6) **** | 815 ± 298  (n=9) **** |

**Supplementary Table 1:** Endothelial cell density (cells/mm², EM-3000, Tomey Corp., Nagoya, Japan) in eyes with granular corneal dystrophy (GCD), lattice corneal dystrophy (LCD) and macular corneal dystrophy (MCD) after penetrating keratoplasty. The endothelial cell density (mean ± standard deviation) are shown postoperatively after 6 weeks to 6 months (6w-6m), 1 year (y), after 1^st^ and 2^nd^ suture removal, and then annually up to more than 5 years (5y+). The preoperative baseline values were compared with the postoperative values within a group. Significant values are highlighted with asterisks (*** p≤0.001 / **** p≤0.0001).
